# Acute exercise alters immune responses in older adults, with extracellular vesicle changes observed in a high-intensity intervention

**DOI:** 10.3389/fimmu.2025.1661161

**Published:** 2025-10-24

**Authors:** Alejandra P. Garza, Lorena Morton, Anna-Lena Motsch, Christian Puta, Marvin Stiebler, Yves Lading, Sabyasachi Chakrabarty, Stefanie Schreiber, Edit I. Buzás, Rüdiger Braun-Dullaeus, Patrick Müller, Ildiko R. Dunay

**Affiliations:** ^1^ Institute of Inflammation and Neurodegeneration, Otto-von-Guericke University, Magdeburg, Germany; ^2^ Department of Sports Medicine and Health Promotion, Friedrich-Schiller-University Jena, Jena, Germany; ^3^ Department for Internal Medicine IV (Gastroenterology, Hepatology and Infectious Diseases), Jena University Hospital, Jena, Germany; ^4^ Center for Sepsis Control and Care (CSCC), Jena University Hospital/Friedrich-Schiller-University Jena, Jena, Germany; ^5^ Department of Cardiology and Angiology, University Hospital Magdeburg, Magdeburg, Germany; ^6^ Center for Behavioral Brain Sciences (CBBS), Magdeburg, Germany; ^7^ Department of Neurology, University Hospital Magdeburg, Magdeburg, Germany; ^8^ German Center for Neurodegenerative Diseases (DZNE), Magdeburg, Germany; ^9^ Department of Neurology, Heinrich Heine University Düsseldorf, Düsseldorf, Germany; ^10^ Department of Genetics, Cell- and Immunobiology, Semmelweis University, Budapest, Hungary; ^11^ HCEMM SU Extracellular Vesicle Research Group, Budapest, Hungary; ^12^ HUN-REN-SU Translational Extracellular Vesicle Research Group, Budapest, Hungary; ^13^ German Center for Mental Health (DZPG), Magdeburg, Germany; ^14^ Centre for Intervention and Research on Adaptive and Maladaptive Brain Circuits Underlying Mental Health (C-I-R-C), Magdeburg, Germany

**Keywords:** cardiovascular fitness, healthy aging, inflammaging, extracellular vesicles, sex differences

## Abstract

**Introduction:**

Aging is accompanied by immunoscenescence and chronic low-grade inflammation (inflammaging), contributing to age-related diseases. Physical exercise is a potent modulator of immune function and systemic inflammation, yet the effects of acute exercise intensity on immune activation, cytokine dynamics, and extracellular vesicle release in older adults remain incompletely characterized, particularly in a sex-specific context. This study investigated how a single session of acute continuous moderate versus intense exercise modulates immune cell subsets, cytokine levels, and EV profiles in healthy older individuals, with emphasis on sex-based differences.

**Methods:**

Thirty-three older adults completed either a moderate (n=14, 54-79 years; 60% VO_2_max, 30 minutes) or an intense cycling bout (n=19, 61-85 years; incremental cardiopulmonary exercise test (CPET) to exhaustion). Peripheral blood was collected at baseline, 30 minutes, and 24 hours post-exercise. Immune cells were analyzed by flow cytometry. EVs were characterized by flow cytometry and nanoparticle tracking analysis, and cytokines were quantified by multiplex assays.

**Resuls:**

Moderate exercise enhanced classical monocyte activation (↑CD86, ↓CX3CR1) without altering cell counts, and selectively elevated IL-6 in females. Intense exercise induced stronger innate immune activation, increasing classical and nonclassical monocytes, CD56^bright^/CD16^low^ NK cells, and sustained TNFα levels. EVs positive for tetraspanins (CD9, CD63, and CD81) were elevated 24h after intense CPET. Exploratory sex-disaggregated analyses revealed distinct profiles: females had increased CD4^+^ EVs, while males showed elevated HLA-ABC^+^ EVs.

**Discussion:**

Acute exercise modulates immune responses in an intensity- and sex-dependent manner in older adults. Extracellular vesicle release was assessed only in the high-intensity intervention, where significant changes were observed. These findings support personalized exercise regimens to enhance immune resilience and promote healthy aging.

## Graphical Abstract

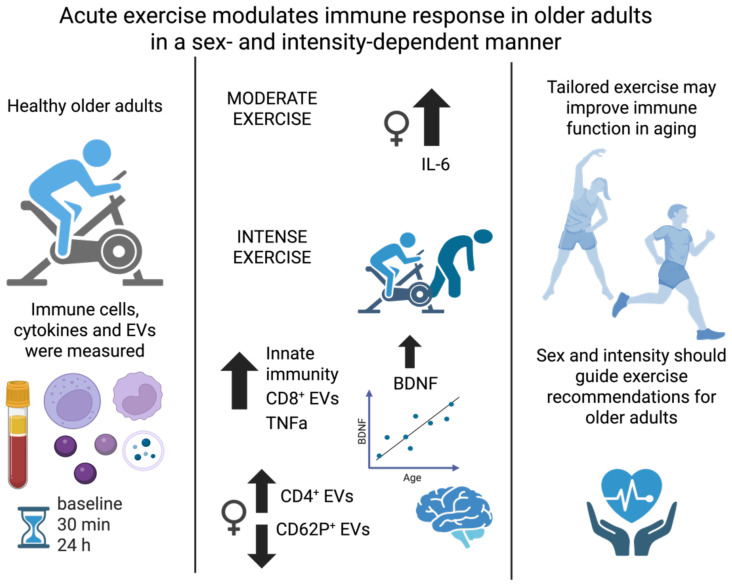

## Highlights

- Immunosenescence and inflammaging underscore the importance of exploring exercise as a therapeutic strategy in older adults.
- This study explores how acute moderate and intense exercise modulates peripheral immune responses in aging.
- Exercise triggers extracellular vesicles release and immune activation, with distinct responses between sexes.
- Further studies should clarify sex-specific immune responses to exercise to inform personalized aging interventions.

## Background

Aging is characterized by a progressive decline in physiological function and increased susceptibility to chronic diseases such as cardiovascular (e.g., heart failure, chronic coronary syndrome), metabolic (e.g., Diabetes Mellitus 2) and neurodegenerative (e.g., dementia) diseases, as well as cancer (1–6). Age-related immune changes, or immunosenescence, contribute to heightened disease vulnerability and impaired infection response (7–9). Chronic low-grade inflammation, or *inflammaging*, is a hallmark of aging and contributes to the pathogenesis of age-related diseases (10, 11). As the global population ages, understanding the complex interplay between exercise, immune function, inflammation, and aging is essential to promote healthy aging and reduce the burden of age-related diseases (12, 13).

Physical activity confers numerous health benefits and is essential for promoting healthy aging. While evidence supports that exercise positively impacts outcomes and prognoses across numerous diseases in the elderly (14, 15), the underlying mechanisms of exercise-induced release, uptake, and communication of bioactive factors in older adults remain incompletely understood. This is especially true regarding sex-specific responses as males and females may exhibit distinct immune adaptations to exercise. Regular exercise modulates immune function by reducing systemic inflammation markers and attenuates age-related increases in proinflammatory cytokine levels, making it a powerful approach for mitigating inflammaging and mitigate chronic disease risk in older adults (10, 13, 16–19). Importantly, sex-specific differences in immune function suggest that personalized exercise regimens could optimize immune responses and overall health outcomes, highlighting the need for targeted approaches in exercise prescriptions for aging populations (20, 21).

Age-related immune changes include alterations in immune cell composition and function, dysregulation of cytokine signaling, and impaired immune surveillance (22–26). While the benefits of physical exercise on immune function are well-documented, the precise mechanisms driving these effects, particularly in the older adult population, remain incompletely understood.

In recent years, extracellular vesicles (EVs), particularly those released in response to exercise (ExerVs), have emerged as a subject of significant interest due to their role in facilitating intercellular communication. By transporting proteins, lipids, and microRNAs to distant organs, EVs act as carriers of bioactive molecules potentially mediating exercise-induced benefits across the organism. Evidence suggests that exercise modulates EV release into circulation pointing toward a pathway through which physical activity may influence immune responses in aging populations (27–32).

Despite growing recognition of the importance of physical exercise in healthy aging, major gaps remain in our understanding of the mechanisms underlying its effects on immune function, inflammation, and aging, along with sex-related discrepancies (10, 18, 33). While most studies emphasize long-term training adaptations, acute exercise represents the fundamental unit of physical activity. Acute exercise has long been recognized as a potent stimulus for immune surveillance, mobilizing NK cells, T cells, and neutrophils into circulation (34). Moderate bouts enhance immunosurveillance and anti-inflammatory signaling, whereas prolonged strenuous exercise may transiently suppress immune function (35). Acute immune and EV responses are thought to accumulate over time, shaping the trajectory of healthy aging and adaptation.

Thus, our research aimed to investigate these gaps by examining the effects of acute continuous moderate and intense exercise interventions on immune responses, cytokine levels, and the release of EVs in older adults. Exercise intensity is a key determinant of these effects: moderate activity is well tolerated and clinically recommended for older adults, whereas intense bouts may trigger more pronounced but potentially less sustainable immune responses. Direct comparison allows us to assess how intensity shapes immune and EV responses, informing exercise prescriptions for older adults. We selected 30 min and 24 h after exercise as sampling points to capture early post-exercise changes and subsequent recovery. Although additional intermediate measures (e.g., at peak or 3–4 h) might reveal further dynamics, these timepoints balanced mechanistic insight with practical considerations in older adults, including participant burden and study logistics. Furthermore, we aimed to explore the relevance of sex-specific responses, focusing on how tailored exercise interventions may promote immune resilience and health in aging populations. Together, these factors guided our investigation of how acute exercise intensity shapes immune and EV responses in older adults.

## Methods

### Study population

A cohort of 33 healthy older individuals (subproject 1: 14 participants, 54–79 years; 60% VO_2_max, 30 minutes; subproject 2: 19 participants, 61–85 years; incremental cardiopulmonary exercise test (CPET) to exhaustion), recruited through the University Hospital Magdeburg, participated in this study. The inclusion criteria were age > 55 years and the ability to move freely. Participants with a history of cardiovascular, endocrinological, neurological, neoplastic, or psychiatric disorders were excluded. While the term ‘older adults’ is used throughout this manuscript, we acknowledge that this is a relative term; in our study, it refers to individuals aged 55 years and above.

### Intervention

#### Subproject 1

**Figure 1:**
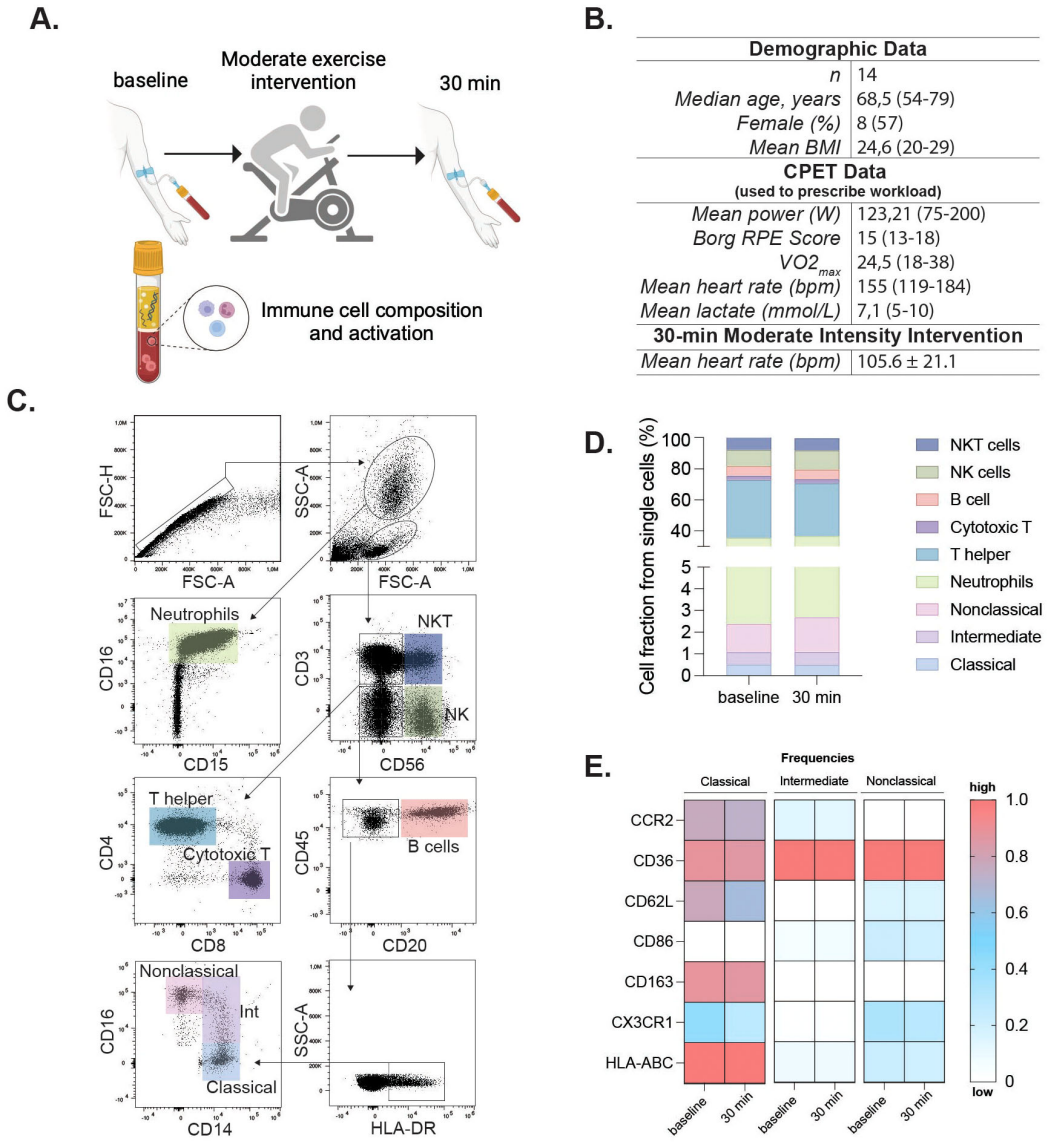
Moderate exercise alters immune activation in older adults. Clinical and experimental study design and analytical processes for the moderate exercise intervention. Participants completed a cycling cardiopulmonary exercise testing (CPET) for 30 minutes; peripheral blood was assessed at baseline and 30 min after the intervention **(A)**. Demographic and exercise data for participants in the moderate intervention. VO_2_max and performance parameters (mean power, RPE, peak heart rate, and peak lactate) were obtained during the cardiopulmonary exercise test (CPET) used to prescribe the 60% VO_2_max workload for the 30-min cycling intervention. Mean heart rate (105.6 ± 21.1 bpm) reflects values recorded during the 30-min moderate-intensity intervention itself. **(B)**. Flow cytometry gating identified single cells, granulocytes, and mononuclear cells via FSC/SSC. Neutrophils were further gated by co-expression of CD16/CD15. Mononuclear cells were gated on CD3 and CD56 to define NKT (CD3^+^CD56^+^) and NK (CD3^-^CD56^+^) cells. CD3^-^CD56^-^ cells were assessed for CD20 (B cells), and CD20^-^ cells further gated by CD14 and CD16 for classical (CD14^+^CD16^-^), intermediate (CD14^+^CD16low), and nonclassical (CD14lowCD16^++^) monocytes **(C)**. Immune cell fractions are shown in bar charts **(D)**. Heatmaps showing frequencies of activation marker-positive cells within classical, intermediate, and nonclassical monocyte subsets at baseline and 30min post-exercise. Rows represent markers, columns represent timepoints and subsets. Colors indicate relative frequency (red = higher, blue = lower) **(E)**. Statistical significance was assessed using non-parametric tests. Panel **(A)** was created in BioRender (Garza, AP. 2024, https://BioRender.com/o96f904).

Fourteen healthy adults (median age: 68 years, 57% female) underwent a moderate acute continuous exercise intervention on a bicycle ergometer (30 minutes, 60% VO_2_max). Individual cardiorespiratory fitness was assessed at least two weeks earlier using a cardiopulmonary exercise test (CPET) to exhaustion, and the workload for the intervention was calculated as 60% of each participant’s measured VO_2_max. The mean Vo_2_max of participants in this group was 24.5 mL/kg/min (range 18–38) (**Figures 1A, B**). Blood was collected before and 30 minutes after the intervention (**Figure 1A**).

#### Subproject 2

**Figure 2:**
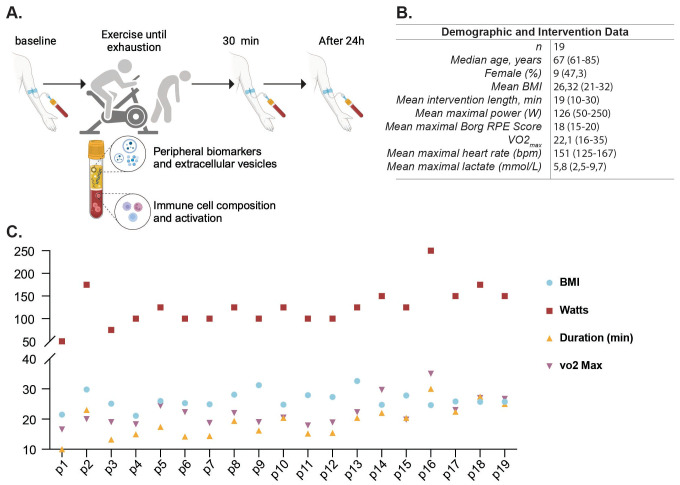
Study design and participant characteristics for intense exercise intervention. Clinical and experimental study design and analytical processes for the intense exercise intervention. Participants completed an incremental cycling cardiopulmonary exercise testing (CPET) to volitional exhaustion; peripheral blood was assessed at baseline, 30 min, and 24 h after the intervention **(A)**. Demographic and intervention metrics for the cohort (sex distribution, age, body mass index (BMI), intervention duration, peak power (W), VO_2_max, Borg RPE score, maximal heart rate and lactate) **(B)**. Individual participant data for BMI, Watts reached, duration of the intervention in minutes, and VO_2_max. **(C)**. Panel A was created in BioRender (Garza, AP. 2024, https://BioRender.com/c87a107).

Nineteen healthy adults (median age: 67 years, 47% female) performed a cardiopulmonary exercise test until exhaustion on a bicycle ergometer. The incremental step test included a three-minute unloaded pedaling at 0 W, following the resistance increased by 25 W every three minutes. During the incremental cycling test, breath-by-breath pulmonary gas-exchange data (MetaSoft, Studio: Cortex Biophysik GmbH Leipzig, Germany), heart rate (Custo med 100, custo med GmbH, Ottobrunn, Germany) and lactate levels (Lactate Scout 4, EKF Diagnostic, Barleben, Germany) were assessed. Perceived exertion was assessed at the end of each step using Borg-Scale (Borg, 1970). The CPET concluded when (i) the respiratory exchange ratio was above 1.10, (ii) a plateau in VO_2_ occurred (despite increasing workload) or (iii) the rating of perceived exertion was 18 or higher on the Borg Scale. Safety criteria for premature termination of CPET were in the case of major electrocardiographic abnormalities, excessive blood pressure increase (≥ 230 mmHg systolic and/or ≥ 110 mmHg diastolic), or individual request (30). The mean VO_2_max in this group was 22.1 mL/kg/min (range 16–35) (**Figures 2B, C** for individual values). Blood samples were collected at baseline, 30 min and 24 hours post-intervention (**Figure 2A**).

### Blood collection and processing

Whole blood was obtained from the antecubital area via venipuncture using a 21G butterfly needle in sterile BD Vacutainer blood collection tubes containing 1 mL Acid Citrate Dextrose/Glucose (ACD). Samples were processed within 1 h of collection. Whole blood (100 µL) was lysed using 1X Red Blood Cell Lysis Buffer (BioLegend, 10X) following the manufacturer’s instructions. After lysis, cells were centrifuged at 400 × g for 5 min at room temperature. The supernatant was discarded, and two washing steps were performed using PBS. Cells were resuspended in 300 µL of FACS buffer (1X PBS, 2% FBS, 2 mM EDTA, and 2 mM NaN3), and 100 µL was added to a 5 mL round-bottom polystyrene tube. Samples were incubated in 5 µL of Human TruStain FcX (BioLegend) for 10 min to avoid nonspecific antibody binding. Each sample was stained with the following antibodies: anti-human CD16 (FITC), anti-human HLA-DR (Peridinin chlorophyll protein-Cyanine5.5), anti-human CD86 (Allophycocyanin), anti-human CD3 (Alexa Fluor 700), anti-human CD66b (Alexa Fluor 700), anti-human CD19 (Alexa Fluor 700), anti-human CD56 (Alexa Fluor 700), anti-human CD36 (Allophycocyanin-Cyanine 7), anti-human CD163 (Brilliant Violet 421), anti-human CD15 (Brilliant Violet 510), anti-human HLA-ABC (Brilliant Violet 605), anti-human CCR2 (Phycoerythrin), anti-human CD62L (Phycoerythrin -Dazzle 594), anti-human CD15 (Phycoerythrin-Cyanine5), anti-human CX3CR1 (Phycoerythrin-Cy7), anti-human CD4 (Allophycocyanin-Cyanine 7), anti-human CD3 (Brilliant Violet 510), anti-human CD16 (Brilliant Violet711), anti-human CD45 (FITC), anti-human CD56 (Phycoerythrin), anti-human CD8 (Phycoerythrin-Dazzle 594) and anti-human CD20 (Peridinin chlorophyll protein-Cyanine5.5). After an incubation period of 30 min, the samples were washed twice and resuspended in 210 µL of FACS buffer. Samples were acquired using an Attune NxT flow cytometer (Thermo Fisher Scientific).

### Gating strategy

Data were first gated on singlets (FSC-H vs FSC-A) and on size/complexity (FSC-A vs SSC-A) to exclude doublets and debris. Within this population, neutrophils were identified as CD15^+^CD16^+^ cells in the high-SSC gate. From the lower SSC/FSC region, lymphocyte populations were further defined: NK cells as CD3^-^CD56^+^, NKT cells as CD3^+^CD56^+^, and T cells as CD3^+^ events subdivided into CD4^+^ helper and CD8^+^ cytotoxic subsets. B cells were identified as CD3^-^CD56^-^CD20^+^. Monocytes were gated as CD20^-^CD45^+^ cells, followed by selection for HLA-DR^+^ events, and then classified by CD14/CD16 expression into classical (CD14^+^CD16^-^), intermediate (CD14^+^CD16^low^), and nonclassical (CD14^low^CD16^++^) subsets. Fluorescence Minus One controls (FMOs) were used to define gates, and single-stained compensation beads were used to calculate compensation. Data were analyzed using FlowJo (v10.10.0).

### Cytokine assay

Plasma was separated from whole blood by centrifugation at 1,500 g for 10 min. Plasma (1 mL) was transferred to clean Eppendorf tubes and centrifuged at 400 × g for 10 min at 4 °C to remove debris. The panel included cytokines, chemokines, and soluble factors with established relevance for aging, inflammation, and exercise immunology, encompassing pro-inflammatory (e.g., TNF-α, IL-6), anti-inflammatory (e.g., IL-10, IL-33), vascular and neurotrophic mediators (e.g., VEGF, BDNF, β-NGF, VILIP-1), and soluble immune receptors (e.g., sRAGE, sTREM1, sTREM2). Analytes were assessed using the Human LEGENDplex Multiplex Assay (BioLegend) including TNF-α, IL-6, VEGF, BDNF, IL-23, IL-1β, sRAGE, IL-12p70, IL-18, IL-10, IFN-α2, sTREM2, IFN-γ, β-NGF, CX3CL1, IL-33, VILIP-1, IL-17A, MCP-1, IL-8, and sTREM1; following the manufacturer’s instructions. The assay utilizes allophycocyanin-coated beads conjugated with surface antibodies that allow the specific binding of the analyte of interest. After incubation of the capture beads with the plasma sample, biotinylated detection antibodies were added to create a bead-analyte-detection antibody sandwich, which was further stained with streptavidin-phycoerythrin. Samples were measured using an Attune NxT flow cytometer and analyzed using the LEGENDplex Data Analysis Online Software Suit. Half of the limit of detection (LOD) was used as a constant value when the predicted concentrations were below the LOD.

### Extracellular vesicles

ACD blood samples from the intense exercise intervention group were centrifuged twice at 2,500 g for 20 min at RT, followed by 2 centrifugation cycles of 14,000 g for 70 min at 4 °C. The supernatant was carefully removed, and the pellet was resuspended in 200 µL 0.22 m filtered phosphate-buffered saline (PBS) without Ca^2+^ and Mg^2+^ and vortexed. Thirty-seven EV surface epitopes were studied using the MACSPlex Human Exosome Kit (Miltenyi) following the manufacturer’s instructions, as previously described (36, 37). In short, this assay employs phycoerythrin and fluorescein isothiocyanate-labeled polystyrene capture beads that capture plasma EVs during overnight incubation. Subsequently, allophycocyanin-labeled anti-CD9, anti-CD63, and anti-CD81 antibodies were added to positively select for EVs. This results in the formation of a structure encompassing capture beads, EVs, and detection antibodies, facilitating event detection and identification of surface epitopes. For each sample, raw bead-associated signal (MFI) for every epitope was background-corrected by subtracting the corresponding blank/isotype signal. To account for between-sample differences in total EV capture, per-sample normalization was performed by scaling each epitope’s MFI to the tetraspanin signal measured in the same well, as commonly applied in bead-based EV assays. Samples were measured using an Attune NxT flow cytometer, normalized values were further analyzed using FlowJo (v10.10.0).

### Nanoparticle tracking analysis

NanoSight Pro instrument (Malvern, Worcestershire, UK) was used to determine the size distribution and concentration of the EVs. All the EVs samples were diluted in fPBS (1:1000) and then injected into the sample-carrier cell. The particles were automatically tracked and sized using Brownian motion to record five videos of 30 *s*. The parameters were set at 25 °C, 5 µL/min flow rate, 39 frame rate and exposure of 25 *ms*. The cell was cleaned with fPBS followed by ethanol between samples. The video images were analyzed by the Nanosight Pro 1.2.0.3 software (Malvern Panalytical Inc.) The size mode (nm) and concentration (particle number/mL) of the EVs were calculated by combining the data from the five records. To effectively represent the distribution of particle sizes, we utilized a Kernel Density Estimation (KDE) approach with a shaded deviation region to highlight variability.

### Statistical analyses

Statistical analyses were performed using GraphPad Prism 10 (v10.1.1(270)). Data distribution was assessed with D’Agostino & Pearson and Shapiro-Wilk tests. Differences between two timepoints were assessed using the Wilcoxon matched pairs signed rank test. Mixed-effects analysis, with Geisser-Greenhouse correction and Tukey’s multiple comparison test was performed for the comparison of three time points. Sex-specific analyses were conducted to determine whether the changes observed in the overall population were also present when analyzed separately by sex. Graphical data representations were created using GraphPad Prism 9, BioRender and Phyton. An alpha value of *p* < 0.05 was used for all statistical tests. Statistical significance was set at *p* < 0.05 and marked with asterisks as follows: * *p* < 0.05, ** *p* < 0.001, and *** *p* < 0.0001. Data are presented as the arithmetic mean and corresponding standard error of the mean (SEM).

## Results

### Subproject 1

#### Acute moderate continuous exercise intervention affects monocyte activation 30 min post exercise in older adults

To investigate the acute effect of moderate exercise, 14 participants completed a 30 min cycling intervention at 60% VO_2_max, prescribed on the basis of CPET performed at least 2 weeks earlier (**Figures 1A, B**). During the intervention, participants reached a mean heart rate of 105.6 ± 21.1 bpm. Flow cytometric analysis of whole blood revealed no significant alterations in cell composition or frequency of adaptive or innate immune populations following acute continuous moderate-intensity exercise (**Figures 1C, D**, **Table 1**). However, specific changes were observed in the monocyte subsets. Classical monocytes exhibited increased frequency of CD86 (pre, 20.12 ± 2.83%; post, 26.97 ± 2.70%, *p* = 0.010) and decreased CX3CR1 expression post-intervention (pre, 54.27 ± 2.37%; post, 46.81 ± 3.30%, *p* = 0.034). Intermediate monocytes displayed decreased CX3CR1 expression (pre, 80.12 ± 3.11%; post, 73.40 ± 4.52%, *p* = 0.034) and reduced HLA-ABC levels (pre, 100 ± 0.11%; post, 99.36 ± 0.19%, *p* = 0.021). No discernible changes in the nonclassical subset were observed (**Figure 1E**). Inflammatory cytokines showed no significant differences between baseline and post-intervention groups (**Supplementary Figure 1**).

**Table 1:** Immune cell counts pre- and post-exercise intervention.

n	Baseline	30 min	T-test
	14	14	
	Mean of events per ul (SEM)		P-value
NKT cells	33.25 (4.5)	34.03 (5.4)	0.9598
NK cells	46.74 (5.2)	53.4 (6.9)	0.4879
B cells	27.2 (3.4)	26.0 (3.0)	0.8036
Cytotoxic T cells	12.1 (1.5)	11.7 (5.3)	0.9189
T helper cells	163.7 (23.8)	145.9 (17.3)	0.6027
Neutrophils	146.9 (15.4)	146.6 (8.6)	0.3380
Classical Monocytes	2.3 (0.2)	2.14 (0.2)	0.4542
Intermediate Monocytes	2.51 (0.3)	2.59 (0.3)	0.8300
Nonclassical Monocytes	5.71 (0.4)	6.87 (0.7)	0.3064

Values are presented in events/uL, showing mean and standard error of the mean (SEM) per cell group within each group. Counts are reported as events/µL to enable direct comparison of circulating cell numbers independent of leukocyte frequency; complementary frequencies are presented in **Figure 1D**. Statistical evaluation was performed using student’s t test, *p* values are shown within the table.

#### Sex specific activation of monocyte subsets and IL-6 post exercise response

**Figure 3:**
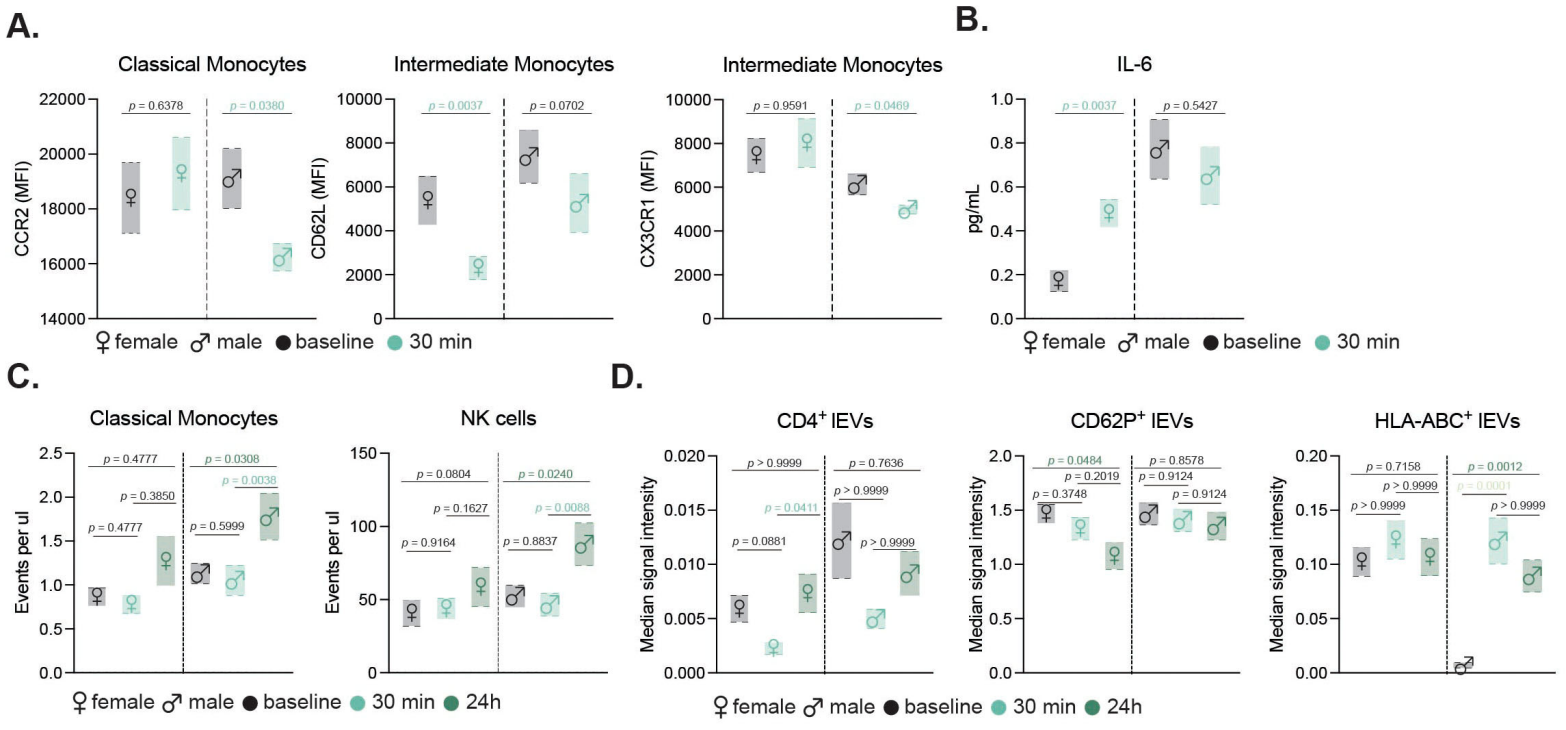
Sex-specific immune and EV responses to exercise in older adults. Floating bar charts showing sex-disaggregated mean ± SEM for monocyte activation markers **(A)** and IL-6 levels **(B)** before and 30 min after moderate exercise. Sex-specific counts of classical monocytes and NK cells **(C)**, and median signal intensity of CD4^+^, CD62P^+^, and HLA-ABC^+^ EVs at baseline, 30 min, and 24 h post-intense exercise **(D)**. Female and male data are shown with respective symbols (♀ left, ♂ right). Statistical tests included non-parametric t-tests (moderate, n=14) and mixed-effects analysis with Geisser-Greenhouse correction and Tukey’s test (intense, n=19). p values are indicated in the figure.

Due to the scarcity of sex-disaggregated data on exercise interventions in healthy older adult populations, we aimed to investigate potential sex-specific differences in the immune response. Given the limited number of participants per group, these analyses are underpowered and should be considered exploratory. While overall immune cell counts between male and female participants did not differ significantly, when examining the activation of monocyte subsets, our results showed sex-specific variations following moderate exercise (**Figure 3A**). Male participants exhibited a notable decrease in CCR2 expression in classical monocytes and a significant increase in CXC3CR1 levels in intermediate monocytes. In contrast, female participants displayed reduced CD62L expression in intermediate monocytes. Additionally, only female participants exhibited an increase in circulating IL-6 levels post-exercise indicating a sex-specific inflammatory response to moderate exercise (**Figure 3B**).

### Subproject 2

#### Acute high-intensity exercise intervention affects monocyte, NK cell and neutrophil changes 24 h post exercise in older adults

To evaluate the effects of an acute high intensity exercise session, 19 healthy participants, with a median age of 67 years and a near-equal distribution of sex (47.3% females), underwent a maximum-intensity cardiopulmonary exercise test (CPET). The acute high-intensive exercise intervention was designed to push participants to their physical limits, enabling the assessment of immune responses and extracellular vesicle release under maximal stress conditions (**Figure 2A**). Participants exhibited a mean BMI of 24.6 and a mean power output of 126 Watts (ranging from 50-250W) during the intervention, which displayed substantial inter-individual variability (**Figure 2B**). The exercise duration and VO_2_max displayed notable similarities among participants, revealing the diverse fitness levels within the cohort (**Figure 2C**). All participants performed the same CPET protocol.

**Figure 4:**
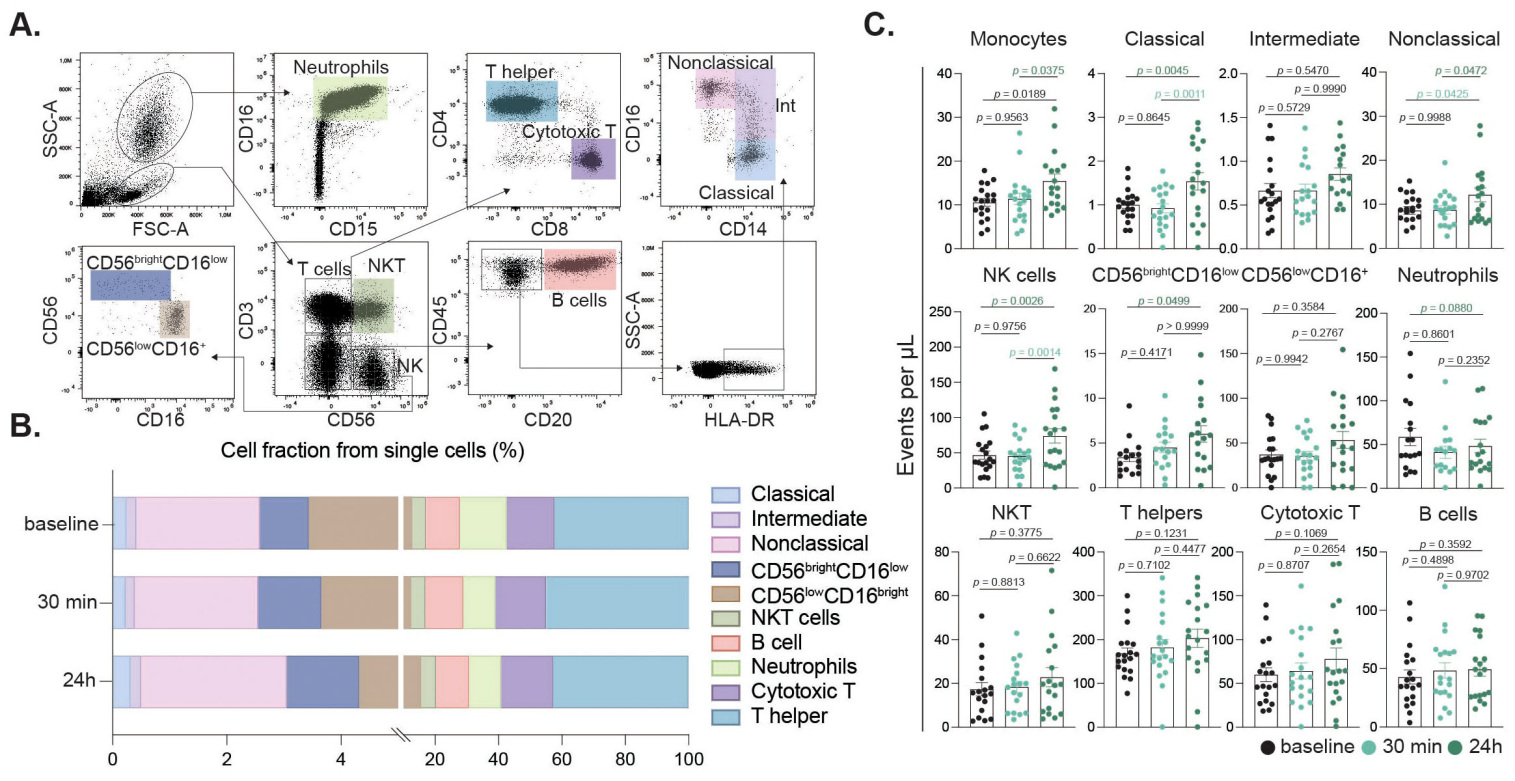
Peripheral immune cell subset alterations induced by intense exercise. Flow cytometry gating identified singlets, granulocytes, and mononuclear cells based on SSC/FSC. Neutrophils were gated by CD16/CD15 expression. Mononuclear cells were defined by CD3/CD56 to distinguish NKT (CD3^+^CD56^+^) and NK (CD3^-^CD56^+^) cells. NK cells were further subdivided into CD56^bright^CD16^low^/CD56^low^CD16^+^ subsets. CD3^-^CD56^-^ cells were assessed for CD20 (B cells); CD20^-^ cells were gated on CD14/CD16 for classical (CD14^+^CD16^-^), intermediate (CD14^+^CD16^low^), and nonclassical (CD14^low^CD16^++^) monocytes **(A)**. Immune cell fractions relative to total single cells at baseline, 30 min, and 24 h post-exercise are shown **(B)**. Bar charts showing cell fractions of immune cell populations at baseline (black), 30 min (turquoise) and 24 h (dark green) after the intervention **(C)**.

To evaluate the impact of intense exercise on immune cell populations, we conducted flow cytometric analysis was performed on blood samples collected at baseline, 30 minutes post-intervention and 24 h post-exercise (**Figure 2A**). This gating strategy allowed for detailed examination of neutrophils, T helper cells, cytotoxic T cells, NK cells, B cells, and monocyte subsets (**Figure 4A**). Our results revealed significant changes in the cellular fractions at the different time points, with the most prominent difference observed at 24 h post-intervention (**Figure 4B**). Specifically, our findings indicated a significant increase in classical and nonclassical monocytes, along with a rise in NK cells driven by CD56^bright^ and CD16^low^ NK cells contributing to the increase (**Figure 4C**). In contrast, neutrophil counts declined 24 h after the intervention, while components of the adaptive immunity remained unchanged (**Figure 4C**). Further sex-disaggregated analysis revealed that the increase in classical monocytes and NK cells was predominantly driven by the male participants, whereas no significant changes were observed in the female subgroup (**Figure 3C**).

#### Monocyte and neutrophil activation after acute intense exercise

**Figure 5:**
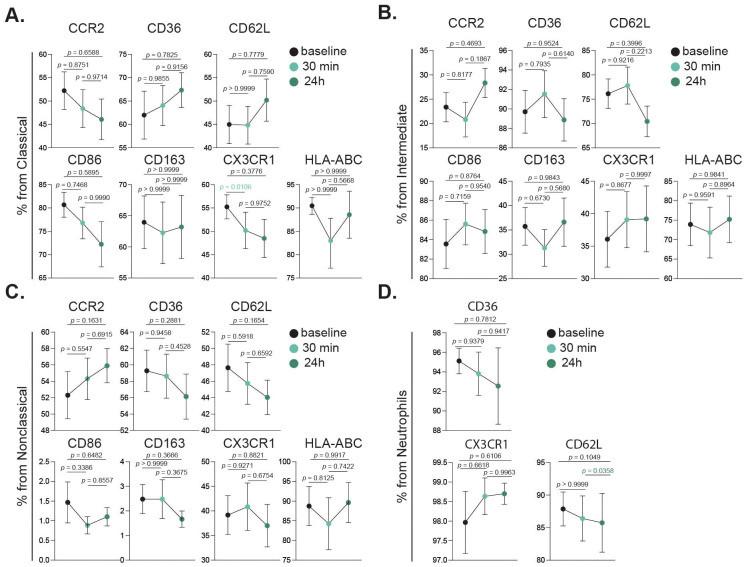
Activation dynamics of innate immune cell subsets following intense exercise. Bar charts showing cell fractions of all investigated activation markers in classical monocytes **(A)**, intermediate monocytes **(B)**, nonclassical monocytes **(C)** and neutrophils **(D)**, at baseline (black), 30 min (turquoise) and 24 h (dark green) after the intervention. Statistical tests analyzed using a mixed-effects analysis, with Geisser-Greenhouse correction and Tukey’s multiple comparison test. *p* values are shown in figure.

Monocyte and neutrophil activation after acute intense exercise was investigated to determine if their activation status was influenced by the intervention. The expression levels of key activation markers including CCR2, CD36, CD62L, CD86, CD163, CX3CR1, and HLA-ABC were evaluated across each monocyte subset. In classical monocytes (**Figure 5A**), CX3CR1 expression was significantly reduced 30 min post intervention. In contrast, intermediate (**Figure 5B**) and nonclassical (**Figure 5C**) monocytes did not exhibit significant changes in the studied markers. For neutrophils, a significant decrease in CD62L expression was observed between the 30 min and 24 h time points (**Figure 5D**).

#### Elevated proinflammatory cytokine levels following acute high intensity exercise

**Figure 6:**
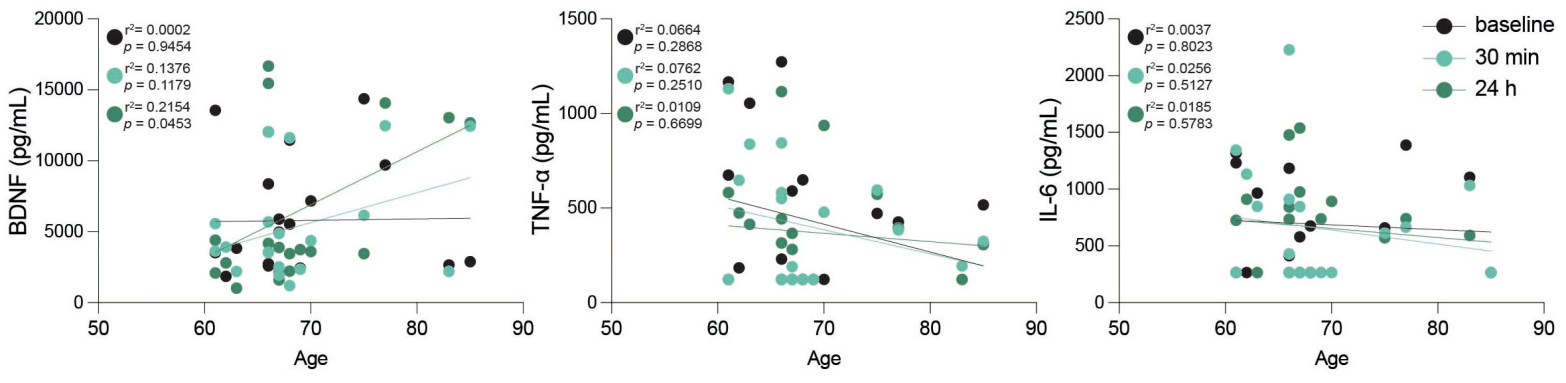
Peripheral BDNF correlates with age after intense exercise. Scatter plots including linear regression lines displaying peripheral levels of brain-derived neurotrophic factor (BDNF), tumor necrosis factor-alpha (TNF-α), and interleukin-6 (IL-6) in relation to age across the three studied time points: baseline (black), 30 min post-exercise (turquoise), and 24 h post-exercise (dark green). Data points represent individual participants, with black, turquoise and dark green colors indicating different time points as specified. r^2^ and p values are shown in plot; statistical analysis was performed using simple linear regression.

Aging is linked to elevated levels of inflammatory cytokines, creating an environment conducive to age-related diseases (38). Additionally, it has been shown that intense acute exercise in young individuals typically triggers a surge in canonical proinflammatory cytokines (39), and we aimed to determine if similar responses occur in an older adult cohort. Therefore, to assess the inflammatory impact of high-intensity exercise, we measured various cytokines and regulatory biomolecules at baseline, 30 minutes and 24 hours post-intervention (**Table 2**). Our study revealed that high-intensity exercise significantly increased peripheral TNFα levels immediately post-exercise (5.32 pg/mL) compared to baseline (2.68 pg/mL, p = 0.047), and remained elevated 24 h post-intervention (4.23 pg/mL), evidencing a sustained inflammatory response (**Table 2**). IL-6 levels did not show significant changes across time points, although a slight decrease was observed at 24 h post-intervention. Similarly, markers associated with vascular and brain health, such as sRAGE, sTREM2, and β-NGF, did not exhibit significant changes. However, a separate analysis showed a significant correlation of BDNF levels with age 24 h after the continuous acute intense exercise intervention (**Figure 6**). Anti-inflammatory cytokine IL-10 showed a transient non-significant decrease immediately post-intervention returning to baseline levels by 24 h. Other proinflammatory cytokines (IL-1β, IL-12p70, IL-18, IL-23, IL-17A, IFN-γ) and chemokines (MCP-1, IL-8, CX3CL1) showed no significant changes across timepoints. Likewise, anti-inflammatory mediators (IL-10, IL-33) and soluble receptors (sTREM1, sTREM2, sRAGE) remained stable. Neurotrophic and vascular markers (VEGF, β-NGF, VILIP-1) also did not differ significantly between timepoints (**Table 2**).

**Table 2:** Circulating cytokine levels in the intense exercise intervention.

n	Baseline	30 min	24 h	ANOVA
	19	19	19	
	pg/mL (m, IQR)			p-value
TNFa	2.68 (0.23)	5.32 (43.14)	4.23 (11.57)	0.0470
IL-6	4.96 (6.74)	4.63 (8.42)	2.64 (3.5)	0.0927
VEGF	3.79 (11.53)	4.16 (10.69)	8.1 (26.13)	0.1154
BDNF	252.8 (392)	325.7 (499.6)	276.7 (244)	0.1322
IL-23	3.95 (6.18)	9.66 (18.68)	7.00 (11.59)	0.1694
IL-1b	0.33 (3.79)	0 (0.79)	0 (0.17)	0.1881
sRAGE	2536 (4082.3)	1988 (2140)	2414 (3240)	0.2236
IL-12p70	4.72 (4.52)	5.87 (10.80)	3.77 (7.27)	0.2349
IL-18	191.7 (76.7)	199.5 (154.9)	202.4 (122)	0.3974
IL-10	5.90 (16.69)	3.23 (6.57)	4.49 (6.43)	0.4509
IFN-a2	3.08 (5.36)	4.45 (9.17)	4.24 (7.14)	0.5177
sTREM2	3736 (3489)	4484 (3133)	4450 (3890)	0.5390
IFN-y	0.74 (1.88)	0.65 (1.14)	0.97 (2.77)	0.6022
β-NGF	2.25 (6.73)	1.25 (1.19)	2.67 (4.30)	0.6374
CX3CL1	731.9 (965.6)	836 (1331.5)	1121 (1135.6)	0.7603
IL-33	100.8 (89.1)	109 (143.9)	95.65 (171)	0.7603
VILIP-1	27.05 (212.32)	20.95 (46.35)	88.65 (157.37)	0.8102
IL-17A	0.93 (1.34)	0.75 (1.49)	1.02 (1.23)	0.8487
MCP-1	24.7 (22.83)	25.57 (24.11)	27.22 (25.6)	0.9071
IL-8	8.34 (44.18)	6.54 (30.36)	0.50 (49.81)	0.9081
sTREM1	44.6 (90.76)	29.29 (51.62)	46.17 (118.55)	0.9161

Values are presented in pg/mL, showing median and inter-quartile range (IQR) per analyte within each group. Statistical evaluation was performed using mixed-effects analysis, with Geisser-Greenhouse correction and Tukey’s multiple comparison test, was performed for the comparison of baseline, 30 min, and 24 h after the intense exercise intervention.

#### Increased extracellular vesicle release is a key response to intense exercise

**Figure 7:**
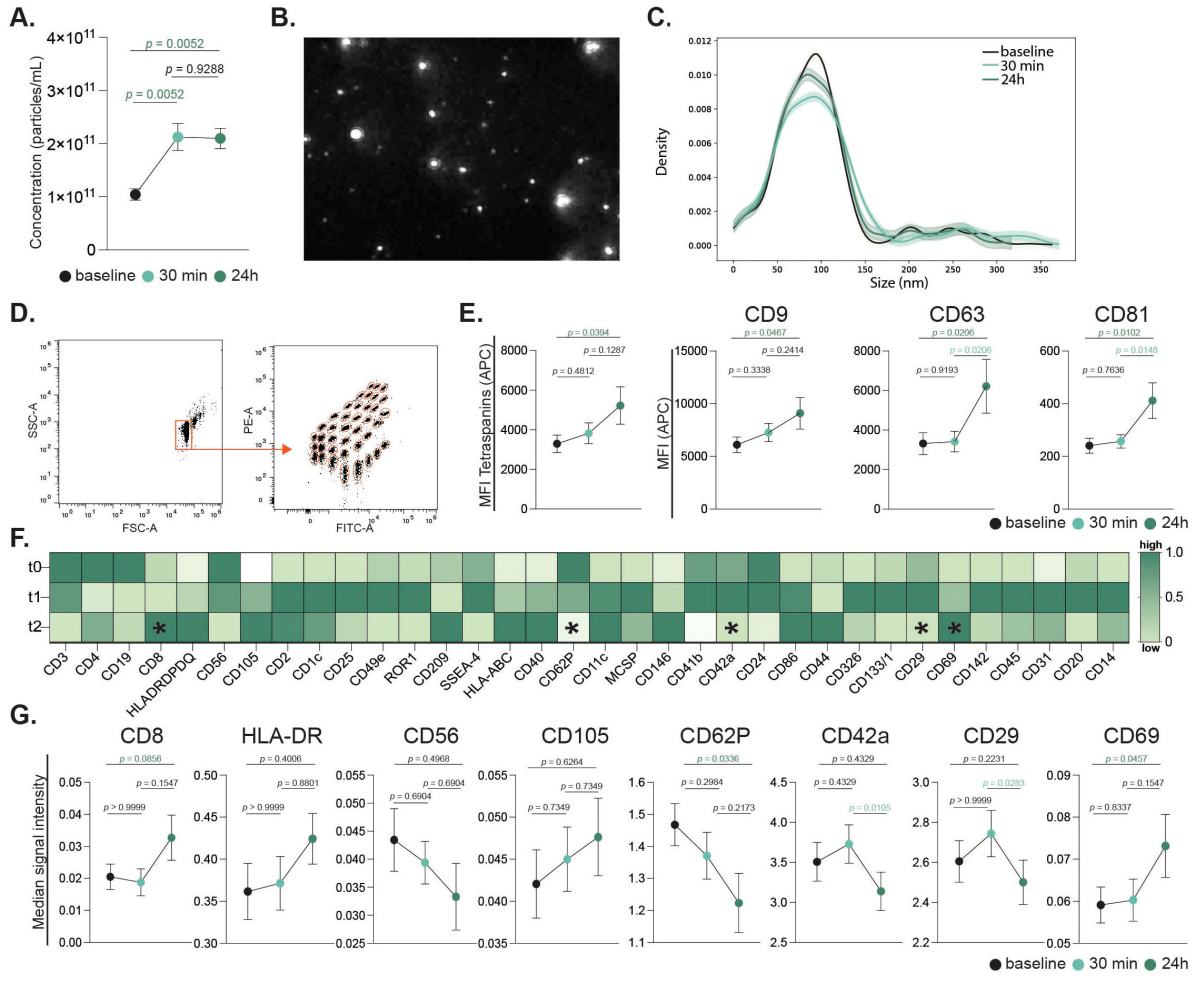
Extracellular vesicles release in response to acute continuous intensive exercise. Plasma-derived EV concentrations were quantified by nanoparticle tracking analysis (NTA) across all timepoints **(A)**. Representative EV imaging **(B)** and size distribution histogram are shown **(C)**. Bead-based flow cytometry assessed 37 EV surface markers, with representative gating and fluorescence profiles **(D)**. Tetraspanin expression (CD9, CD63, CD81) is presented as mean ± SEM and individual values **(E)**. Heatmap showing normalized mean fluorescence intensity (MFI) of 34 differentially expressed markers scaled to tetraspanin expression (CD9, CD63 and CD81). Rows represent timepoints (pre-, 30 min, and 24 h post-exercise); columns represent markers. Colors indicate relative expression (dark green = higher, light green/white = lower). Asterisks denote significant changes between timepoints **(F)**. Signal intensities of selected immune, endothelial, and platelet-derived markers (CD8, HLA, CD56, CD105, CD62P, CD42a, CD29, CD69) are shown **(G)**. Statistical analysis used mixed-effects modeling with Geisser-Greenhouse correction and Tukey’s test. p values are indicated in the figure.

Following the evaluation of immune cell populations, their activation status, and peripheral cytokine levels, we examined the impact of high-intensity exercise on the release of EVs. Plasma-derived EVs were quantified at baseline, 30 minutes and 24 h post-intervention. Nano-particle tracking analysis (NTA) showed an increased concentration of EVs after intense exercise at both studied timepoints (**Figures 7A–C**). Flow cytometric analysis demonstrated a general increase in the concentration of plasma-derived EVs after 24 hours, as indicated by increased levels of tetraspanin markers CD9, CD63, and CD81 (**Figures 7D, E**). Notably, CD63 and CD81 displayed significant increases in median fluorescence intensity (MFI) at 24 h compared to 30 minutes post-intervention, while CD9 levels remained unchanged. Since our EV profile was limited to surface epitope analysis, the findings should be interpreted as descriptive, and future studies should address functional properties and cargo composition.

A detailed examination of 37 exosomal surface epitopes revealed a significant increase in surface CD69 expression after 24 h post-intervention (**Figure 7F**). This increase in CD69, a marker of cellular activation, was accompanied by a significant reduction in CD62P, CD42a, and CD29 markers on EVs at 24 h post-intervention. Other markers including CD8, CD56, and CD105, did not show significant changes across the time points (**Figure 7G**). Sex-specific differences were also observed in EV profiles. While the total circulating EV count did not differ between sexes, females exhibited a significant increase in CD4+ EVs and a decrease in CD62P+ EVs 24 hours post-exercise. In contrast, male participants showed a marked increase in HLA-ABC+ EVs following the intense exercise intervention (**Figure 3D**).

## Discussion

In recent years, the *Exercise as Medicine* approach has been increasingly recognized for its multifaceted benefits beyond disease prevention (40). Exercise acts as a modulator of immune function and inflammation, key elements in aging and disease processes (33, 41, 42). Training adaptations are ultimately built on repeated acute bouts of exercise. Characterizing immune and EV responses to a single session would provide a mechanistic insight into how physical activity contributes to healthy aging over time. Therefore, our study aimed to explore the interplay between exercise and the immune response, revealing alterations in immune cell populations and cytokine levels following both acute continuous moderate and intense exercise interventions in older individuals. Furthermore, we examined exercise induced release and selective surface marker modulation of EVs as potential mediators of systemic immune response, contributing to a growing body of evidence that exercise plays a vital role in promoting healthy aging and preventing age-related diseases. Additionally, sex-stratified responses were explored. These findings, while intriguing, are based on small subgroup sizes and should be viewed as exploratory until confirmed in larger cohorts.

### Monocyte subsets activation after moderate exercise

We examined the impact of a single session of acute continuous moderate exercise on the peripheral innate immune system of older adults. Although overall cell numbers and distributions were unchanged, activation patterns of monocyte subsets differed. Prior work shows that monocyte responses depend on exercise intensity (43). Our findings align with this, as classical (CD14^+^CD16^-^) monocytes, representing an early differentiation stage are recruited during moderate aerobic exercise (in our study acute continuous moderate exercise: 30 minutes, 60% VO_2_max, subproject 1). In contrast, mature subtypes (non-classical and intermediate monocytes, CD14^+^CD16^+^) increase predominantly after high-intensity exercise (CPET to exhaustion, subproject 2). Since aging is associated with chronic innate immune activation and expansion of CD16^+^ monocytes, these observations highlight exercise as a potential intervention target for healthy aging (44).

Classical monocytes, which are key to phagocytosis and leukocyte recruitment, displayed increased CD86 and decreased CX3CR1 expression. CD86, a co-stimulatory molecule rapidly mobilized from intracellular stores, supports enhanced T cell co-stimulation (45). Reduced CX3CR1, a chemokine receptor regulating monocyte differentiation and migration, mirrors prior findings that its ligand CX3CL1 rises in skeletal muscle after exercise to promote a regenerative microenvironment (46). Intermediate monocytes also showed decreased CX3CR1, consistent with peripheral differentiation, accompanied by an overall increase in intermediate and nonclassical monocytes (47). Furthermore, we observed a significant reduction in CCR2 on classical monocytes, restricted to older males. CCR2, a key chemokine receptor for monocyte recruitment, has shown sex-specific patterns in younger adults, with females generally expressing higher levels post-exercise (48, 49). In our cohort, older females exhibited higher baseline CCR2 than males, but only males showed a post-exercise reduction. This suggests sex-dependent modulation of monocyte trafficking, potentially shaped by hormonal influences, and highlights the value of considering sex in personalized exercise prescriptions for immune health in aging.

Intermediate monocytes also showed reduced HLA-ABC expression, a class I MHC antigen involved in antigen presentation. While microglial HLA-ABC expression increases with age and may contribute to vulnerability to structural brain changes (50, 51), its reduction in circulating monocytes could indicate a transient state of altered immune surveillance. In addition, we observed a decrease in CD62L, an adhesion molecule required for monocyte rolling and endothelial transmigration (52, 53). Prior studies report higher CD62L expression in older adults compared to younger cohorts (54). Interestingly, in our study, this reduction was specific to females, suggesting enhanced adhesion and transmigration capacity post-exercise. In contrast, the relatively stable CD62L levels observed in males may reflect less efficient monocyte trafficking, potentially contribute to higher chronic inflammation and the greater risk of cardiovascular disease in men.

### Circulating pro-inflammatory cytokines after one moderate session of exercise in older adults

Prior studies report increases in IL-6 and TNF-α after moderate exercise (39, 55, 56), but we observed no cytokine changes 30 min post-exercise. The broader cytokine and biomarker panel also remained stable, suggesting that moderate exercise did not elicit a systemic inflammatory response in this cohort. Participants reported an average Borg RPE of 15 (range 13-18), indicating effort levels theoretically sufficient to provoke cytokine release (57). The absence of detectable increases may reflect sampling time or participants regular engagement in physical activity. Notably, IL-6 was increased selectively in females. Prior work links sex hormones to IL-6 dynamics, with women often exhibiting higher IL-6 than men under stress or ischemic conditions (58). In older adults, IL-6 also correlates with sarcopenia in a sex-dependent manner (59). Our data therefore suggests that moderate aerobic exercise may elicit distinct inflammatory responses in older women versus men, underscoring the need to consider sex when evaluating exercise-induced immune effects in aging populations. While these sex-specific effects are intriguing, they should be interpreted with caution given the small subgroup sizes, and future studies with larger cohorts are needed to confirm them.

### Intense exercise modifies the innate immune response

Exercise-induced leukocyte mobilization is well documented, and is partly attributed to cortisol-mediated release from the bone marrow (60, 61). We hypothesized that an intense exercise would exert stronger effects than moderate exercise, and indeed observed enhanced innate responses, particularly in NK cells and monocytes 24h post-intervention. In line with previous work identifying NK cells as the most responsive subset (39, 62–65), increases were mainly driven by CD56^bright^ and CD16^low^ NK cells. Intense exercise also induced a sustained rise in TNFα, likely reflecting cytokine production from activated NK cells (66, 67).

In contrast, other cytokines including IL-6, IL-10, IL-23, IL-12p70, IL-18, chemokines (MCP-1, IL-8, CX3CL1), and neurotrophic/vascular factors such as BDNF, VEGF, β-NGF, and soluble receptors (sRAGE, sTREM1, sTREM2), remained largely unchanged suggesting that the cytokine signature of intense exercise in older adults is relatively restricted. Further studies with larger cohorts are needed to fully elucidate the broader immunological impact of intense physical activity in older individuals.

Classical and nonclassical monocytes also increased at 24h, accompanied by reduced CX3CR1 expression, consistent with a shift toward a proinflammatory phenotype. Neutrophils count declined at 24h, likely reflecting recruitment to damaged muscle tissue, as reported in animal models (68, 69). This aligns with previous findings by Nunes-Silva et al. who reported that exhaustive treadmill exercise in mice induced rolling, adhesion, and transmigration of neutrophils into muscle tissue (70). Supporting this, neutrophils in our cohort exhibited increased CX3CR1 expression, a mechanism shown to facilitate muscle infiltration during exercise (71).

BDNF, a neurotrophic factor consistently elevated by acute and long-term exercise (72–74) showed a transient increase 30 min post-intervention, returning toward baseline at 24h. Notably, BDNF correlated positively with age at 24h, suggesting that older individuals exhibit a more prolonged response. This may represent an age-related adaptation in BDNF sensitivity or a compensatory neuroprotective mechanism. Whether this delayed response translates into functional benefits such as improved recovery or neuroplasticity warrants further study.

### Extracellular vesicle release after exercise in older adults

EVs mediate many of the beneficial effects of physical activity by transporting proteins, lipids, and nucleic acids that shape intercellular communication and metabolism (75–78). Prior work in younger athletes showed exercise-induced EV release primarily from lymphocytes, monocytes, platelets, endothelial cells, and MHC-II+ cells (79). In our older cohort, both sexes exhibited increases in EVs derived from lymphocytes and leukocytes (CD8, CD14, HLA, CD86), platelets (CD62P, CD42a, CD41b), and endothelial cells (CD105, CD31), consistent with these earlier findings (30, 79). We also observed increased CD69 expression on EVs 24h post-exercise, reflecting leukocyte activation in parallel with NK cell and monocyte mobilization (80). Platelet markers (CD62P, CD42a) were increased at 30 min but declined by 24h suggesting transient platelet activation that may contribute to recovery processes in aging individuals.

Although total numbers did not differ by sex, distinct patterns emerged. Females showed an increase in CD4^+^ T cell-derived EVs, whereas males exhibited higher HLA-ABC+ EVs 24h after exercise. These findings suggest sex-dependent adaptive immune modulation not apparent at the cellular level, potentially influenced by hormonal factors such as estrogen, which enhances CD4^+^ T cell activity, or testosterone, which promotes antigen presentation (81). Physiological adaptations may also contribute, with females exhibiting stronger immune modulation and males favoring recovery and resolution pathways. While the mechanisms remain speculative, our data indicate that exercise-induced EV responses may vary in a sex-dependent manner, underscoring the need for future research. Importantly, as our EV analyses relied on surface epitope profiling, functional properties and cargo composition should be addressed in future studies.

## Conclusions and limitations

Physical exercise impacts several physiological systems beyond solely skeletal muscle, with growing recognition on its role in promoting healthy aging (13, 82). In our study, acute moderate and intense exercise in older adults altered immune cell subsets, cytokine production and EV release. These findings support the concept that tailored exercise prescriptions, accounting for individual and sex-specific responses, may optimize immune resilience and health outcomes in aging populations. EV responses, however, were only observed following high-intensity exercise, and thus our conclusions regarding exercise intensity and EV modulation should be interpreted with caution. The observed modulation of EV surface markers highlights their potential as biomarkers and possible mediators of exercise-induced immune effects, warranting further investigation into their functional roles.

At the same time, these results should be viewed as exploratory. The sample size was relatively small, particularly for sex-stratified analyses, limiting power to detect subtle effects. The parallel-group design, rather than a crossover, prevented within-subject comparisons across intensities. Participants were healthy, active older adults of predominantly European background, which may not generalize to sedentary individuals, those with chronic disease, or more diverse populations. Although VO_2_max values were comparable between groups, we cannot exclude the influence of baseline cardiorespiratory fitness on the immune responses observed. In addition, although the moderate bout was prescribed at 60% VO_2_max, relative intensity defined by VO_2_max may not always correspond to traditional heart rate or lactate thresholds. This divergence has been noted previously in adults, including older cohorts, where VO_2_max-based and threshold-based measures of intensity show only modest concordance and substantial interindividual variability in older populations (83, 84). Blood was collected at three timepoints, but additional sampling could provide a more complete temporal profile of immune and EV dynamics. Finally, EV profiling was restricted to surface epitope analysis, and potential confounders such as diet, sleep or hormonal status were not systematically controlled.

Taken together, our findings indicate that both moderate and intense exercise induce measurable changes in immune cell activation in older adults. In addition, EV release was assessed in the high-intensity intervention, where significant changes were observed. Preliminary evidence suggests potential sex-specific differences. While limited in scope, these results underscore the value of integrating exercise into healthy aging strategies and highlight the need for larger, more diverse, and mechanistically focused studies to fully define the therapeutic potential of exercise as medicine.

## Data Availability

The raw data supporting the conclusions of this article will be made available by the authors, without undue reservation.

## Supplementary material

Supplementary Figure 1 Peripheral cytokine levels 30 minutes following acute continuous moderate exercise. Violin plots display plasma concentrations of selected cytokines in older adults, measured at baseline and 30 minutes after a session of moderate-intensity exercise. Plots showing the central line representing the median value, and thinner lines above and below the median mark the interquartile range (IQR). Statistical evaluation was performed using student’s t test, *p* values are shown in figure.

## Glossary

ACD: Acid Citrate Dextrose/Glucose
APC: Allophycocyanin
BDNF: Brain-derived Neurotrophic Factor
BMBF: Federal Ministry of Education and Research
BMI: Body Mass Index
CD: Cluster of Differentiation
CPET: Cardiopulmonary Exercise Testing
EDTA: Ethylenediaminetetraacetic acid
EV: Extracellular Vesicle
ExerVs: Exercise-released Extracellular Vesicles
FACS: Flow Cytometry
FBS: Fetal Bovine Serum
FITC: Fluorescein
fPBS: Filtered Phosphate Buffered Saline
FSC: Forward Scatter
FSC-A: Forward Scatter – Area
HLA: Human Leukocyte Antigens
IL: Interleukin
IQR: Inter-quartile Range
KDE: Kernel Density Estimation
LOD: Limit of Detection
MFI: Median Fluorescence Intensity
MHC-I: Major Histocompatibility Complex Class I Antigen
NGF: Nerve Growth Factor
NK: Natural Killer
NKT: Natural Killer T cells
NTA: Nanoparticle tracking analysis
PBMC: Peripheral Blood Mononuclear Cells
PBS: Phosphate Buffered Saline
PE: R-Phycoerythrin
RPE: Rate of Perceived Exertion
SEM: Standard Error of the Mean
SSC: Side Scatter
SSC-A: Side Scatter - Area
sTREM2: Soluble Triggering Receptor Expressed on Myeloid Cells 2
TNF: Tumor Necrosis Factor
VO_2_: Volume of Oxygen
VO_2_max: Maximum Volume of Oxygen

## References

[1] Hiam-Galvez KJ Allen BM Spitzer MH. Systemic immunity in cancer. Nat Rev Cancer. (2021) 21:6. doi: 10.1038/s41568-021-00347-z, PMID: 33837297 PMC8034277

[2] Lutshumba J Nikolajczyk BS Bachstetter AD. Dysregulation of systemic immunity in aging and dementia. Front Cell Neurosci. (2021) 15:652111/BIBTEX. doi: 10.3389/FNCEL.2021.652111, PMID: 34239415 PMC8258160

[3] Zmora N Bashiardes S Levy M Elinav E. The role of the immune system in metabolic health and disease. Cell Metab. (2017) 25:506–21. doi: 10.1016/J.CMET.2017.02.006, PMID: 28273474

[4] Boyalla V Gallego-Colon E Spartalis M. Immunity and inflammation in cardiovascular disorders. BMC Cardiovasc Disord. (2023) 23:148. doi: 10.1186/S12872-023-03185-Z, PMID: 36959565 PMC10035189

[5] Morton L Arndt P Garza AP Henneicke S Mattern H Gonzalez M. Spatio-temporal dynamics of microglia phenotype in human and murine cSVD: impact of acute and chronic hypertensive states. Acta Neuropathol Commun. (2023) 11. doi: 10.1186/S40478-023-01672-0, PMID: 38115109 PMC10729582

[6] Triposkiadis F Xanthopoulos A Butler J. Cardiovascular aging and heart failure: JACC review topic of the week. J Am Coll Cardiol. (2019) 74:804–13. doi: 10.1016/J.JACC.2019.06.053, PMID: 31395131

[7] Wang Y Dong C Han Y Gu Z Sun C. Immunosenescence, aging and successful aging. Front Immunol. (2022) 13:942796/BIBTEX. doi: 10.3389/FIMMU.2022.942796/BIBTEX 35983061 PMC9379926

[8] Liberale L Badimon L Montecucco F Lüscher TF Libby P Camici GG. Inflammation, aging, and cardiovascular disease: JACC review topic of the week. J Am Coll Cardiol. (2022) 79:837–47. doi: 10.1016/J.JACC.2021.12.017, PMID: 35210039 PMC8881676

[9] Ungvari Z Tarantini S Sorond F Merkely B Csiszar A. Mechanisms of vascular aging, A geroscience perspective: JACC focus seminar. J Am Coll Cardiol. (2020) 75:931–41. doi: 10.1016/J.JACC.2019.11.061, PMID: 32130929 PMC8559983

[10] Ferrucci L Fabbri E. Inflammageing: chronic inflammation in ageing, cardiovascular disease, and frailty. Nat Rev Cardiol (2018) 15:505–22. doi: 10.1038/s41569-018-0064-2, PMID: 30065258 PMC6146930

[11] Ginaldi L Di Benedetto MC De Martinis M. Osteoporosis, inflammation and ageing. Immun Ageing. (2005) 2:1–5. doi: 10.1186/1742-4933-2-14/METRICS 16271143 PMC1308846

[12] Mensah GA Habtegiorgis Abate Y Abbasian M Abd-Allah F Abdollahi A Abdollahi M. Global burden of cardiovascular diseases and risks, 1990-2022. J Am Coll Cardiol. (2023) 82:2350–473. doi: 10.1016/J.JACC.2023.11.007, PMID: 38092509 PMC7615984

[13] Müller P Stiebler M Schwarck S Haghikia A Düzel E. Physical activity, aging and brain health. Dtsch Z Sportmed. (2021) 72:327–34. doi: 10.5960/DZSM.2021.506

[14] Galloza J Castillo B Micheo W. Benefits of exercise in the older population. Phys Med Rehabil Clin N Am. (2017) 28:659–69. doi: 10.1016/J.PMR.2017.06.001, PMID: 29031333

[15] Kokkinos P Faselis C Samuel IBH Pittaras A Doumas M Murphy R. Cardiorespiratory fitness and mortality risk across the spectra of age, race, and sex. J Am Coll Cardiol. (2022) 80:598–609. doi: 10.1016/J.JACC.2022.05.031, PMID: 35926933

[16] Estruel-Amades S Camps-Bossacoma M Massot-Cladera M Pérez-Cano FJ Castell M. Alterations in the innate immune system due to exhausting exercise in intensively trained rats. Sci Rep. (2020) 10:1. doi: 10.1038/s41598-020-57783-4, PMID: 31969634 PMC6976645

[17] Lesnak JB Berardi G Sluka KA. Influence of routine exercise on the peripheral immune system to prevent and alleviate pain. Neurobiol Pain. (2023) 13:100126. doi: 10.1016/J.YNPAI.2023.100126, PMID: 37179769 PMC10173010

[18] Weyh C Krüger K Strasser B. Physical activity and diet shape the immune system during aging. Nutrients. (2020) 12(3):622. doi: 10.3390/nu12030622, PMID: 32121049 PMC7146449

[19] Paolucci EM Loukov D Bowdish DME Heisz JJ. Exercise reduces depression and inflammation but intensity matters. Biol Psychol. (2018) 133:79–84. doi: 10.1016/J.BIOPSYCHO.2018.01.015, PMID: 29408464

[20] Klein SL Flanagan KL. Sex differences in immune responses. Nat Rev Immunol. (2016) 16:10. doi: 10.1038/nri.2016.90, PMID: 27546235

[21] Ji H Gulati M Cheng S. ReSex diversity in physical activity benefits: absolute and relative differences in context.ply. J Am Coll Cardiol. (2024) 84:e35–6. doi: 10.1016/j.jacc.2024.04.058, PMID: 39048286

[22] Papp G Szabó K Jámbor I Mile M Berki AR Arany AC. Regular exercise may restore certain age-related alterations of adaptive immunity and rebalance immune regulation. Front Immunol. (2021) 12:639308. doi: 10.3389/fimmu.2021.639308, PMID: 33936054 PMC8085426

[23] van der Geest KSM Wang Q Eijsvogels TMH Koenen HJP Joosten I Brouwer E. Changes in peripheral immune cell numbers and functions in octogenarian walkers - an acute exercise study. Immun Ageing. (2017) 14:1–13. doi: 10.1186/S12979-017-0087-2/FIGURES/4, PMID: 28250797 PMC5322590

[24] Teissier T Boulanger E Cox LS. Interconnections between inflammageing and immunosenescence during ageing. Cells. (2022) 11:359. doi: 10.3390/CELLS11030359, PMID: 35159168 PMC8834134

[25] Wang L Mao L Xiao W Chen P. Natural killer cells immunosenescence and the impact of lifestyle management. Biochem Biophys Res Commun. (2023) 689:149216. doi: 10.1016/j.bbrc.2023.149216, PMID: 37976836

[26] Duggal NA Niemiro G Harridge SDR Simpson RJ Lord JM. Can physical activity ameliorate immunosenescence and thereby reduce age-related multi-morbidity? Nat Rev Immunol. (2019) 19:9. doi: 10.1038/s41577-019-0177-9, PMID: 31175337

[27] Bei Y Xu T Lv D Yu P Xu J Che L. Exercise-induced circulating extracellular vesicles protect against cardiac ischemia-reperfusion injury. Basic Res Cardiol. (2017) 112:38. doi: 10.1007/s00395-017-0628-z, PMID: 28534118 PMC5748384

[28] McIlvenna LC Whitham M. Exercise, healthy ageing, and the potential role of small extracellular vesicles. J Physiol. (2023) 601:4937–51. doi: 10.1113/JP282468, PMID: 35388915 PMC10952297

[29] Chong MC Shah AD Schittenhelm RB Silva A James PF Wu SSX. Acute exercise-induced release of innate immune proteins via small extracellular vesicles changes with aerobic fitness and age. Acta Physiologica. (2024) 240(3):e14095. doi: 10.1111/APHA.14095, PMID: 38243724

[30] Frühbeis C Helmig S Tug S Simon P Krämer-Albers EM. Physical exercise induces rapid release of small extracellular vesicles into the circulation. J Extracell Vesicles. (2015) 4:28239. doi: 10.3402/JEV.V4.28239, PMID: 26142461 PMC4491306

[31] Whitehead M Antonazzi M Shanahan CM. Extracellular vesicles: the key to unlocking mechanisms of age-related vascular disease? J Cardiovasc Aging. (2024) 4. doi: 10.20517/JCA.2023.49

[32] Darragh IAJ Mcnamee N Daly R Pacheco SM O’driscoll L Egan B. The separation and identification of circulating small extracellular vesicles from endurance-trained, strength-trained and recreationally active men. J Physiol. (2023) 601:5075–91. doi: 10.1113/JP285170, PMID: 37725436

[33] Simpson RJ Lowder TW Spielmann G Bigley AB LaVoy EC Kunz H. Exercise and the aging immune system. Ageing Res Rev. (2012) 11(3):404–20. doi: 10.1016/j.arr.2012.03.003, PMID: 22465452

[34] Nieman DC Wentz LM. The compelling link between physical activity and the body’s defense system. J Sport Health Sci. (2019) 8(3):201–17. doi: 10.1016/j.jshs.2018.09.009, PMID: 31193280 PMC6523821

[35] Nieman DC Pence BD. Exercise immunology: Future directions. J Sport Health Sci. (2020) 9(5):432–45. doi: 10.1016/j.jshs.2019.12.003, PMID: 32928447 PMC7498623

[36] Garza AP Morton L Pállinger É Buzás EI Schreiber S Schott BH. Initial and ongoing tobacco smoking elicits vascular damage and distinct inflammatory response linked to neurodegeneration. Brain Behav Immun Health. (2023) 28:100597. doi: 10.1016/j.bbih.2023.100597, PMID: 36817509 PMC9931921

[37] Garza AP Wider-Eberspächer E Morton L Van Ham M Jänsch L Dunay IR. Circulating pre- and postprandial extracellular vesicle proteomic profiles. Sci Rep. (2024) 14:23032. doi: 10.21203/RS.3.RS-4426110/V1 PMC11450010 39363010

[38] Michaud M Balardy L Moulis G Gaudin C Peyrot C Vellas B. Proinflammatory cytokines, aging, and age-related diseases. J Am Med Dir Assoc. (2013) 14:877–82. doi: 10.1016/J.JAMDA.2013.05.009, PMID: 23792036

[39] Bernecker C Scherr J Schinner S Braun S Scherbaum WA Halle M. Evidence for an exercise induced increase of TNF-α and IL-6 in marathon runners. Scand J Med Sci Sports. (2013) 23:207–14. doi: 10.1111/J.1600-0838.2011.01372.X, PMID: 22092703

[40] Martinez MW Kim JH Shah AB Phelan D Emery MS Wasfy MM. Exercise-induced cardiovascular adaptations and approach to exercise and cardiovascular disease: JACC state-of-the-art review. J Am Coll Cardiol. (2021) 78:1453–70. doi: 10.1016/J.JACC.2021.08.003, PMID: 34593128

[41] Simpson RJ Krüger K Walsh NP Campbell JP Gleeson M Nieman DC. Can exercise affect immune function to increase susceptibility to infection? Exerc Immunol Rev. (2020) 26., PMID: 32139352

[42] Via J Reid J Oehlert G. Pedalling toward a deeper understanding of exercise effects on immune function. J Physiol. (2024) 602:4705–7. doi: 10.1113/JP287173, PMID: 39167713

[43] Gabriel H Kindermann W. The acute immune response to exercise: what does it mean? J Sports Med. 26:8–22, PMID: 9129261 10.1055/s-2007-972698

[44] Hearps AC Martin GE Angelovich TA Cheng WJ Maisa A Landay AL. Aging is associated with chronic innate immune activation and dysregulation of monocyte phenotype and function. Aging Cell. (2012) 11:867–75. doi: 10.1111/J.1474-9726.2012.00851.X, PMID: 22708967

[45] Smyth CM Logan G Boadle R Rowe PB Smythe JA Alexander IE. Differential subcellular localization of CD86 in human PBMC-derived macrophages and DCs, and ultrastructural characterization by immuno-electron microscopy. Int Immunol. (2005) 17:123–32. doi: 10.1093/INTIMM/DXH193, PMID: 15623548

[46] Strömberg A Olsson K Dijksterhuis JP Rullman E Schulte G Gustafsson T. CX3CL1–a macrophage chemoattractant induced by a single bout of exercise in human skeletal muscle. Am J Physiol Regul Integr Comp Physiol. (2016) 310:R297–304. doi: 10.1152/AJPREGU.00236.2015, PMID: 26632602

[47] Panek CA Ramos MV Mejias MP Abrey-Recalde MJ Fernandez-Brando RJ Gori MS. Differential expression of the fractalkine chemokine receptor (CX3CR1) in human monocytes during differentiation. Cell Mol Immunol. (2015) 12:6. doi: 10.1038/cmi.2014.116, PMID: 25502213 PMC4716621

[48] She S Ren L Chen P Wang M Chen D Wang Y. Functional roles of chemokine receptor CCR2 and its ligands in liver disease. Front Immunol. (2022) 13:812431. doi: 10.3389/FIMMU.2022.812431, PMID: 35281057 PMC8913720

[49] Blanks AM Pedersen LN Bohmke N Mihalick VL Franco RL. Sex differences in monocyte CCR2 expression and macrophage polarization following acute exercise. Life Sci. (2022) 299. doi: 10.1016/j.lfs.2022.120557, PMID: 35447130

[50] Ginaldi L De Martinis M D’Ostilio A Marini L Loreto F Modesti M. Changes in the expression of surface receptors on lymphocyte subsets in the elderly: Quantitative flow cytometric analysis. Am J Hematol. (2001) 67:63–72. doi: 10.1002/AJH.1082, PMID: 11343377

[51] Lazarczyk MJ Kemmler JE Eyford BA Short JA Varghese M Sowa A. Major Histocompatibility Complex class I proteins are critical for maintaining neuronal structural complexity in the aging brain. Sci Rep. (2016) 6:1. doi: 10.1038/srep26199, PMID: 27229916 PMC4882527

[52] Ivetic A Green HLH Hart SJ. L-selectin: A major regulator of leukocyte adhesion, migration and signaling. Front Immunol. (2019) 10:1068/BIBTEX. doi: 10.3389/FIMMU.2019.01068/BIBTEX, PMID: 31139190 PMC6527602

[53] Merah-Mourah F Cohen SO Charron D Mooney N Haziot A. Identification of novel human monocyte subsets and evidence for phenotypic groups defined by interindividual variations of expression of adhesion molecules. Sci Rep. (2020) 10. doi: 10.1038/s41598-020-61022-1, PMID: 32157175 PMC7064612

[54] Cao Y Fan Y Li F Hao Y Kong Y Chen C. Phenotypic and functional alterations of monocyte subsets with aging. Immun Ageing. (2022) 19. doi: 10.1186/S12979-022-00321-9, PMID: 36514074 PMC9745938

[55] Docherty S Harley R McAuley JJ Crowe LAN Pedret C Kirwan PD. The effect of exercise on cytokines: implications for musculoskeletal health: a narrative review. BMC Sports Science Med Rehabil. (2022) 14:1. doi: 10.1186/S13102-022-00397-2, PMID: 34991697 PMC8740100

[56] Shobeiri P Seyedmirzaei H Karimi N Rashidi F Teixeira AL Brand S. IL-6 and TNF-α responses to acute and regular exercise in adult individuals with multiple sclerosis (MS): a systematic review and meta-analysis. Eur J Med Res. (2022) 27:185. doi: 10.1186/S40001-022-00814-9, PMID: 36156182 PMC9511785

[57] Borg G. Psychophysical scaling with applications in physical work and the perception of exertion. Scand J Work Environ Health. (1990) 16:55–8. doi: 10.5271/sjweh.1815, PMID: 2345867

[58] Sullivan S Hammadah M Wilmot K Ramadan R Pearce BD Shah A. Young women with coronary artery disease exhibit higher concentrations of interleukin-6 at baseline and in response to mental stress. J Am Heart Assoc. (2018) 7. doi: 10.1161/JAHA.118.010329, PMID: 30571600 PMC6405549

[59] Mikó A Pótó L Mátrai P Hegyi P Füredi N Garami A. Gender difference in the effects of interleukin-6 on grip strength - a systematic review and meta-analysis. BMC Geriatr. (2018) 18. doi: 10.1186/S12877-018-0798-Z, PMID: 29739343 PMC5941705

[60] Hansen JB Wilsgård L Østerud B. Biphasic changes in leukocytes induced by strenuous exercise. Eur J Appl Physiol Occup Physiol. (1991) 62:157–61. doi: 10.1007/BF00643735/METRICS, PMID: 2044521

[61] Neves PRDS Tenório TRDS Lins TA Muniz MTC Pithon-Curi TC Botero JP. Acute effects of high- and low-intensity exercise bouts on leukocyte counts. J Exerc Sci Fit. (2015) 13:24–8. doi: 10.1016/J.JESF.2014.11.003, PMID: 29541095 PMC5812872

[62] Pedersen L Idorn M Olofsson GH Lauenborg B Nookaew I Hansen RH. Voluntary running suppresses tumor growth through epinephrine- and IL-6-dependent NK cell mobilization and redistribution. Cell Metab. (2016) 23:554–62. doi: 10.1016/j.cmet.2016.01.011, PMID: 26895752

[63] Lin ML Hsu CC Fu TC Lin YT Huang YC Wang JS. Exercise training improves mitochondrial bioenergetics of natural killer cells. Med Sci Sports Exerc. (2022) 54:751–60. doi: 10.1249/MSS.0000000000002842, PMID: 34935709

[64] Koivula T Lempiäinen S Rinne P Rannikko JH Hollmén M Sundberg CJ. The effect of acute exercise on circulating immune cells in newly diagnosed breast cancer patients. Sci Rep. (2023) 13:1. doi: 10.1038/s41598-023-33432-4, PMID: 37085562 PMC10121717

[65] Quintana-Mendias E Rodríguez-Villalobos JM Gastelum-Arellanez A Cervantes N Carrasco-Legleu CE Espino-Solis GP. The effect of acute physical exercise on natural killer cells populations and cytokine levels in healthy women. Sports. (2023) 11. doi: 10.3390/SPORTS11100189, PMID: 37888516 PMC10611276

[66] Cooper MA Fehniger TA Caligiuri MA. The biology of human natural killer-cell subsets. Trends Immunol. (2001) 22:633–40. doi: 10.1016/S1471-4906(01)02060-9, PMID: 11698225

[67] Poli A Michel T Thérésine M Andrès E Hentges F Zimmer J. CD56bright natural killer (NK) cells: an important NK cell subset. Immunology. (2009) 126:458–65. doi: 10.1111/J.1365-2567.2008.03027.X, PMID: 19278419 PMC2673358

[68] Pyne DB. Regulation of neutrophil function during exercise. Sports Med. (1994) 17:245–58. doi: 10.2165/00007256-199417040-00005, PMID: 8009138

[69] Suzuki K Naganuma S Totsuka M Suzuki KJ Mochizuki M Shiraishi M. Effects of exhaustive endurance exercise and its one-week daily repetition on neutrophil count and functional status in untrained men. Int J Sports Med. (1996) 17:205–12. doi: 10.1055/S-2007-972833, PMID: 8739575

[70] Nunes-Silva A Bernardes PTT Rezende BM Lopes F Gomes EC Marques PE. Treadmill exercise induces neutrophil recruitment into muscle tissue in a reactive oxygen species-dependent manner. An intravital microscopy study. PLoS One. (2014) 9:e96464. doi: 10.1371/JOURNAL.PONE.0096464, PMID: 24798414 PMC4010495

[71] Nyasha MR Chen W Wang H Yaoita F Aoki M Nagatomi R. Effects of CX3CR1 and CXCR2 antagonists on running-dependent intramuscular neutrophil recruitments and myokine upregulation. Am J Physiol Endocrinol Metab. (2023) 324:E375–89. doi: 10.1152/AJPENDO.00196.2022, PMID: 36856190

[72] Szuhany KL Bugatti M Otto MW. A meta-analytic review of the effects of exercise on brain-derived neurotrophic factor. J Psychiatr Res. (2015) 60:56–64. doi: 10.1016/J.JPSYCHIRES.2014.10.003, PMID: 25455510 PMC4314337

[73] Brunelli A Dimauro I Sgrò P. Emerenziani G Pietro, Magi F, Baldari C, et al. Acute exercise modulates BDNF and pro-BDNF protein content in immune cells. Med Sci Sports Exerc. (2012) 44:1871–80. doi: 10.1249/MSS.0B013E31825AB69B, PMID: 22543740

[74] Müller P Rehfeld K Schmicker M Hökelmann A Dordevic M Lessmann V. Evolution of neuroplasticity in response to physical activity in old age: The case for dancing. Front Aging Neurosci. (2017) 9:56/ABSTRACT. doi: 10.3389/FNAGI.2017.00056/ABSTRACT, PMID: 28352225 PMC5348543

[75] Lisi V Senesi G Balbi C. Converging protective pathways: Exploring the linkage between physical exercise, extracellular vesicles and oxidative stress. Free Radic Biol Med. (2023) 208:718–27. doi: 10.1016/J.FREERADBIOMED.2023.09.021, PMID: 37739138

[76] McIlvenna LC Parker HJ Seabright AP Sale B Anghileri G Weaver SRC. Single vesicle analysis reveals the release of tetraspanin positive extracellular vesicles into circulation with high intensity intermittent exercise. J Physiol. (2023) 601:5093–106. doi: 10.1113/JP284047, PMID: 36855276 PMC10953002

[77] Iijima H Wang K D’Amico E Tang W-Y Rogers RJ Jakicic JM. Exercise-primed extracellular vesicles improve cell-matrix adhesion and chondrocyte health. Res Sq. (2023). doi: 10.21203/RS.3.RS-2958821/V1, PMID: 37333349 PMC10274961

[78] Maggio S Canonico B Ceccaroli P Polidori E Cioccoloni A Giacomelli L. Modulation of the circulating extracellular vesicles in response to different exercise regimens and study of their inflammatory effects. Int J Mol Sci. (2023) 24:3039. doi: 10.3390/IJMS24033039/S1, PMID: 36769362 PMC9917742

[79] Brahmer A Neuberger E Esch-Heisser L Haller N Jorgensen MM Baek R. Platelets, endothelial cells and leukocytes contribute to the exercise-triggered release of extracellular vesicles into the circulation. J Extracell Vesicles. (2019) 8:1615820. doi: 10.1080/20013078.2019.1615820, PMID: 31191831 PMC6542154

[80] Cibrián D Sánchez-Madrid F. CD69: from activation marker to metabolic gatekeeper. Eur J Immunol. (2017) 47:946. doi: 10.1002/EJI.201646837, PMID: 28475283 PMC6485631

[81] Papapavlou G Hellberg S Raffetseder J Brynhildsen J Gustafsson M Jenmalm MC. Differential effects of estradiol and progesterone on human T cell activation *in vitro* . Eur J Immunol. (2021) 51:2430–40. doi: 10.1002/EJI.202049144, PMID: 34223649

[82] Stiebler M Müller P Bock M Lechner B Lechner K. Turning back the clock on aging? A perspective on selected mechanisms and therapeutic avenues. Dtsch Z Sportmed. (2021) 72:335–43. doi: 10.5960/DZSM.2021.507

[83] Neves LNS Gasparini Neto VH Araujo IZ Barbieri RA Leite RD Carletti L. Is there agreement and precision between heart rate variability, ventilatory, and lactate thresholds in healthy adults? Int J Environ Res Public Health. (2022) 19:14676. doi: 10.3390/IJERPH192214676, PMID: 36429395 PMC9690603

[84] Panton LB Graves JE Pollock ML Garzarella L Carroll JF Leggett SH. Relative heart rate, heart rate reserve, and VO2 during submaximal exercise in the elderly. J Gerontol A Biol Sci Med Sci. (1996) 51. doi: 10.1093/GERONA/51A.4.M165, PMID: 8680999

