# Supplementary figures and images for: Acute exercise alters immune responses in older adults, with extracellular vesicle changes observed in a high-intensity intervention
Source: Front Immunol. 2025 Oct 24;16:1661161. doi: 10.3389/fimmu.2025.1661161 (PMC12591881; doi:10.3389/fimmu.2025.1661161)

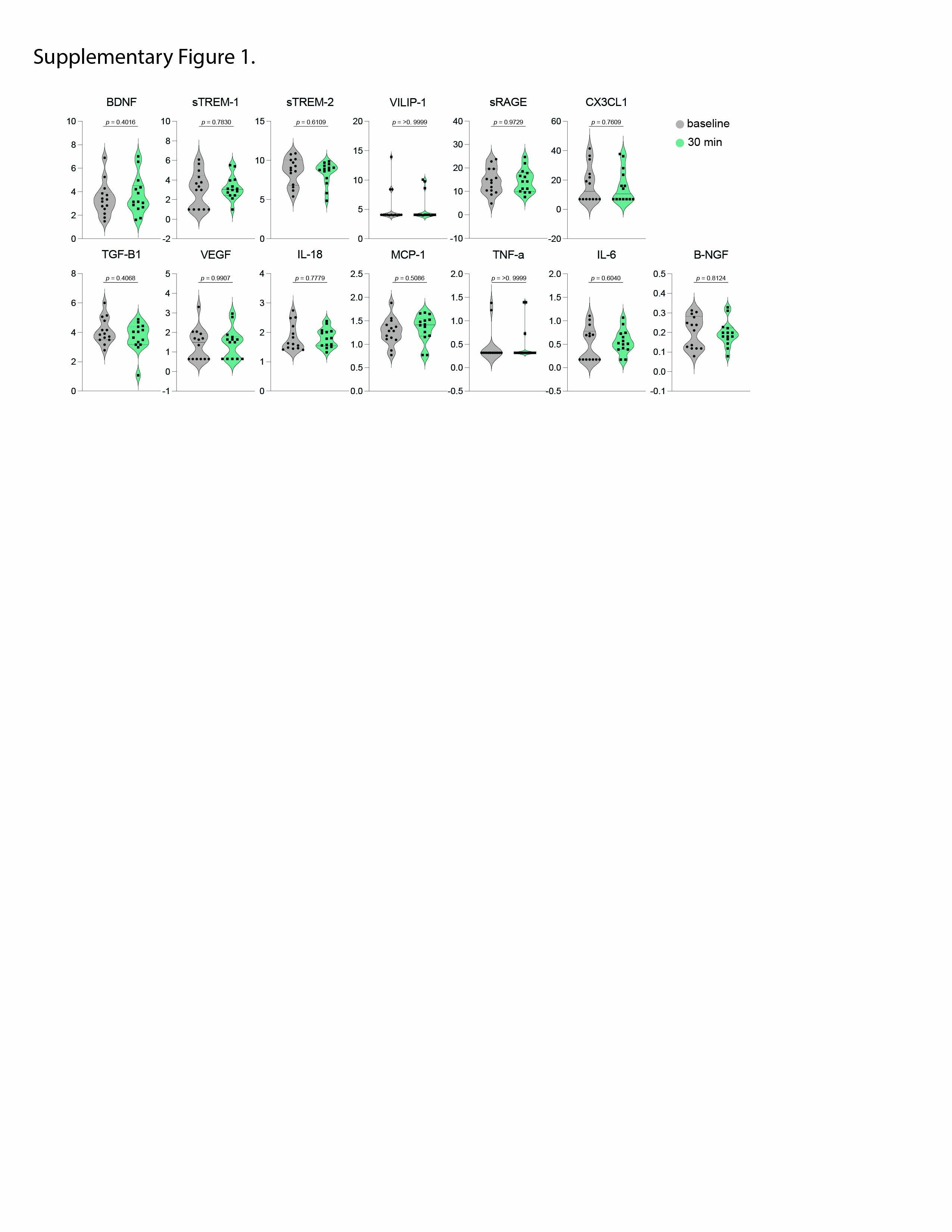

Supplement: Supplementary Figure 1 — Peripheral cytokine levels 30 minutes following acute continuous moderate exercise. Violin plots display plasma concentrations of selected cytokines in older adults, measured at baseline and 30 minutes after a session of moderate-intensity exercise. Plots showing the central line representing the median value, and thinner lines above and below the median mark the interquartile range (IQR). Statistical evaluation was performed using student’s t test, p values are shown in figure. [file Image1.jpeg]
